# WWOX and metabolic regulation in normal and pathological conditions

**DOI:** 10.1007/s00109-022-02265-5

**Published:** 2022-10-22

**Authors:** Izabela Baryła, Katarzyna Kośla, Andrzej K. Bednarek

**Affiliations:** Department of Molecular Carcinogenesis, Medical University of Lodz, Lodz, Poland

**Keywords:** WW domain-containing oxidoreductase WWOX, Steroid metabolism, Lipid metabolism, Glucose metabolism, Bone metabolism

## Abstract

WW domain-containing oxidoreductase (*WWOX*) spans the common fragile site *FRA16D*. There is evidence that translocations and deletions affecting *WWOX* accompanied by loss of expression are frequent in many cancers and often correlate with a worse prognosis. Additionally, *WWOX* germline mutations were also found to be the cause of pathologies of brain development. Because WWOX binds to some transcription factors, it is a modulator of many cellular processes, including metabolic processes. Recently, studies have linked WWOX to familial dyslipidemias, osteopenia, metabolic syndrome, and gestational diabetes, confirming its role as a regulator of steroid, cholesterol, glucose, and normal bone metabolism. The WW domain of WWOX is directly engaged in the control of the activity of transcription factors such as HIF1α and RUNX2; therefore, *WWOX* gene alterations are associated with some metabolic abnormalities. Presently, most interest is devoted to the associations between WWOX and glucose and basic energy metabolism disturbances. In particular, its involvement in the initiation of the Warburg effect in cancer or gestational diabetes and type II diabetes is of interest. This review is aimed at systematically and comprehensively presenting the current state of knowledge about the participation of WWOX in the metabolism of healthy and diseased organisms.

## Introduction

WW domain-containing oxidoreductase (WWOX) spans the common [1] fragile site FRA16D, which is located within a region of frequent loss-of-heterozygosity (LOH) [2, 3]. It is associated with homozygous deletions [1, 2], translocations [3, 4], and other alterations in many cancers [5–7]. More recently, pathogenic variants and mutations in *WWOX* were also described, and these mutations cause a broad range of ultrarare neurodevelopmental and brain degenerative disorders [8], such as WWOX-related epileptic encephalopathy (WOREE syndrome) [9, 10], autosomal recessive spinocerebellar ataxia 12 (SCAR12) [9, 11], and disorder of sex differentiation (DSD) [12, 13]. *WWOX* alterations have also been observed in cases of disorders such as metabolic syndrome [14], gestational diabetes [15], dyslipidemia [16], and osteopenia [24].

The *WWOX* gene encodes a 46-kDa protein that contains two N-terminal WW domains and one short-chain dehydrogenase/reductase (SDR) domain [17]. The WW domains are responsible for protein‒protein interactions and bind proteins sharing PPxY (where P is proline, Y is tyrosine, and x is any amino acid) motifs [18, 19]. Several WWOX protein partners have been recognized, including p73 [20], ErbB4 [21], Ap2α [22] and γ [23], RUNX2 [24], DVL-2 [25], and HIF1α [26]. These interactions show that WWOX is involved in many cellular processes, such as cell proliferation, differentiation, and metabolism. The central SDR domain, whose function is still unknown, is probably responsible for catalyzing the conversion of some low molecular weight ligands, most likely steroids [27].

The first report indicating the role of WWOX in metabolism was a study that used *Wwox*-knockout (KO) mutant mice bred by Aqeilan et al., which suffer from severe metabolic defects that lead to growth retardation and postnatal lethality [28]. At birth, *Wwox* KO pups were identical to WT littermates [24]. However, at 3 days, *Wwox* KO pups were smaller and died by 4 weeks after birth because of severe metabolic defects, mainly hypoglycemia [24, 28]. Researchers observed that *Wwox* KO mice had aberrant serum levels of lipids, carbohydrates, and proteins [24]. Moreover, *Wwox* hypomorphic mice that display reduced levels of *Wwox* expression also had a shorter lifespan than WT mice. However, these mice survived, which is in contrast to the *Wwox* KO mice that showed postnatal lethality [29]. The authors suggested that lower *Wwox* levels are sufficient to overcome the postnatal lethality that was observed in *Wwox* KO mice [29].

## The role of WWOX in steroid metabolism

The amino acid sequence analysis of the SDR (the short-chain dehydrogenase/reductase) domain in WWOX has suggested its role in steroid hormone metabolism [17, 27, 30]. WWOX is highly expressed in hormonally regulated or hormone-secreting organs, such as the prostate, mammary glands, ovaries, and the brain [31]. The high expression in this type of tissue implies that WWOX is probably involved in the modulation of steroid metabolism. In vitro experiments showed that sex steroids, such as estrogen and androgen, lead to the upregulation of WWOX expression [32, 33], and *Wwox* KO mice were characterized by impaired steroidogenesis [34]. Additionally, the mammary-specific KO of *Wwox* in mice disturbed mammary gland development, as defects in mammary branching morphogenesis and ductal outgrowth were observed [35]. Furthermore, in *Wwox* KO mice, defects in the mouse reproductive system were also observed [28]. They are characterized by alterations in the expression of steroidogenic enzymes, impaired levels of steroids, and gonadal dysfunction [28]. Follicle-stimulating and luteinizing hormones were attenuated, and ovaries were significantly smaller in the female *Wwox* KO mouse model than in the control model. Additionally, the testes of *Wwox* hypomorphic males had a high number of atrophic seminiferous tubules and reduced levels of testosterone and fertility disturbance in comparison to their wild-type counterparts [28]. All these data suggest that *WWOX* is associated with gonadal development and plays a crucial role in the normal functioning of hormonally regulated organs and in steroid metabolism.

The growth and survival of early-stage prostate cancers depend on androgens [36, 37]. It was shown that treatment to suppress or block the production or action of male hormones causes them to regress [38]. The absence or reduction of *WWOX* expression has been indicated to be involved in prostate carcinoma [39–41]. Some authors have suggested that the tumor suppressor function of WWOX may be important during the phase of prostate cancer progression when cancer cells shift to be androgen-independent [42]. Additionally, a *Wwox*-deficient mouse model showed that the loss of *Wwox* expression results in testosterone reduction, a condition that may affect prostate functionality. Moreover, it was reported that *WWOX* overexpression induced cell apoptosis and suppressed prostate cancer growth in vitro [22] and in vivo [39].

Apart from the relationship between WWOX and steroid hormone metabolism, the interrelation with estrogen receptors (ER) has also been investigated, especially in breast cancer. A reduction in estrogen receptor expression was observed in the case of *WWOX* KO in the MCF7 ER-positive breast cancer cell line [43], and a positive correlation between *WWOX* expression and ER status was observed in breast cancer patients [42]. The strong relationship between *WWOX* expression and ER status complements the aforementioned evidence for its involvement in sex steroid metabolism.

## The WWOX gene modulates lipid metabolism

Lipid metabolism is the process of synthesis and degradation of lipids in cells [44]. It involves the processes of breakdown or storage of fats to obtain energy and the synthesis of structural and functional lipids. There are three major classes of membrane lipid molecules—phospholipids, cholesterol, and glycolipids [45]. High-density lipoproteins (HDLs) absorb cholesterol and carry it back to the liver, where it is ultimately removed from the body. Higher levels of serum HDL cholesterol (HDL‐C) can lower the risk for heart disease and stroke [46, 47], and the incidence of metabolic syndrome increases as HDL levels decrease [48, 49].

Single-nucleotide variants of the *WWOX* gene were associated with the level of HDL-C [50, 51]. Eight genetic variants in the human *WWOX* gene were significantly associated with low-HDL levels [51]. Initially, an association of region-wide significance between the rs2548861 variant of *WWOX* and low HDL-C levels was identified in families of Mexican and European descent with dyslipidemia [16]. This association was also confirmed in the control population [16]. A population effect of this variant on HDL-C levels was demonstrated in large population-based studies [16]. Furthermore, the longitudinal effect of the rs2548861 variant was observed on HDL-C levels in a prospective cohort followed for more than 20 years [16]. Interestingly, the association between the rs2548861 variant in *WWOX* and HDL levels demonstrated an opposite trend in the Roma population [52]. Lee et al. proposed that the rs2548861 variant alone is not a crucial HDL-C determinant but rather that it is a modifying factor that alters serum HDL-C levels [16]. rs2548861 is located in a conserved region of intron 8 of *WWOX.* rs72790052, rs4462603, and rs5818121 are also cosegregated with low HDL-C levels, suggesting that the location of the functional variants is in intron 5 of *WWOX* [51]. The analysis of a mouse quantitative trait locus (QTL) map to interpret results from a human GWAS for genes associated with plasma HDL‐C levels supports the association between the *WWOX* gene and HDL-C metabolism [53].

As shown in *Wwox*-deficient mouse models, the mRNA and protein levels of apolipoprotein A-I (ApoA-I) and ATP-binding cassette transporter A1 (ABCA1), key regulators of HDL metabolism, are altered [51]. In liver tissue–specific *Wwox* KO (*Wwox* ^hep−/−^) male mice, the decrease in AbcA1 mRNA levels translated to a significant reduction in ABCA1 protein levels, but the mRNA and protein levels of ABCA1 were unchanged in female mice of this model [51]. In addition to mediating the rate-limiting step of HDL biogenesis, ABCA1 is also involved in the regulation of very-low-density lipoprotein (VLDL) production [54]. Thus, it protects the function of pancreatic *β*-cells and insulin secretion by maintaining cholesterol homeostasis [54, 55]. It is worth mentioning that ABCA1 is also associated with glucose uptake by GLUT4 in skeletal muscles [56], and the aberration of ABCA1-regulated phenotypes is recognized in metabolic syndrome patients [54]. A sex effect between segregating *WWOX* variants and low HDL-C levels was also observed in French Canadian families with dyslipidemia [51].

*Wwox* KO mice displayed reductions in the levels of serum lipids, the expression of multiple genes involved in cholesterol homeostasis and hydrolysis, and the biosynthesis of triglycerides and displayed disturbed fatty acid biosynthesis in hepatocytes. Together, these results suggest a global effect of WWOX on lipid metabolism pathways [51]. In all *Wwox* ^hep−/−^ mice, the upregulation of crucial lipid metabolism genes, such as *Angptl4*, *Fasn*, *Pltp*, *Gpam*, and *Lipg*, and the downregulation of *ApoA-I*, *Lpl*, and *Insig2* were observed. These results indicate a disturbance in HDL metabolism in both sexes [51]. Moreover, mice with a tissue-specific ablation of *Wwox* in skeletal muscles showed a decreased HDL/LDL ratio and higher triglycerides and cholesterol levels in comparison to control mice [14].

Furthermore, disturbances of lipid metabolism are likely to have critical matters in the pathogenesis of neurological diseases [57, 58], and the integrity of myelin is affected in numerous lipid metabolism disorders [59]. Myelin is crucial for the proper functioning of the nervous system [60]. It contains an extremely high content of lipids. Myelination requires high levels of fatty acid and lipid synthesis [60]. The uptake of external lipids also plays a central role in myelin sheath formation [60]. The association of WWOX with myelin was investigated in different studies, from rat to mouse models and brain organoids [57, 61–64]. For example, a study with a rat model treated with dopaminergic neurotoxin 1-methyl-4-phenyl-pyridinium (MPP +) which led to developing Parkinson’s disease-like symptoms showed that Wwox was present on the myelin sheaths which remained largely intact after MPP + treatment [62]. What is also interesting, mouse brain RNA-seq database analysis showed the highest Wwox expression levels in progenitor oligodendrocytes with significantly lower levels in mature myelinated oligodendrocytes. This observation allowed the authors to propose that Wwox is involved in the oligodendrocyte differentiation and the myelination process [57]. A magnetic resonance imaging (MRI) of WOREE patients’ brains besides the varying brain structural defects usually shows also reduced myelination [13, 65–68]. Currently, the specific neuronal deletion of murine *Wwox* which generates phenotypes that resemble what is observed in WOREE patients was characterized by a significant decrease in transcript levels of genes involved in myelination in the mouse cortex and hippocampus and reduction of myelinated axons [61]. Another neurodegenerative disorder associated with WWOX, which is among others characterized by multifocal regions of demyelination, is multiple sclerosis (MS). The analysis of white matter areas of postmortem brains from MS patients with progressive disease and control patients showed differences in mature oligodendrocyte sub-clusters and significant downregulations of WWOX in chronic active lesions of MS patients [69, 70]. The recent data show that disturbances of oligodendrocytes and pathology of the myelination process are also associated with Alzheimer’s disease. It has been shown that myelin damage probably may be one of the possible causes leading to the presence of amyloid β (Aβ) plaques and tau hyperphosphorylation [71]. It is worth noting that hippocampal neurons from Alzheimer’s disease patients are characterized by reduced WWOX protein levels compared to controls, and loss of WWOX function has an influence on tau hyperphosphorylation by modulating the activity of the GSK3β, EKR, and JNK kinases and the generation of Aβ aggregation [57, 72]. Interesting is also the mechanism by which WWOX is involved in the myelination. As it was described earlier, WWOX has a global effect on lipid metabolism pathways and maintenance of cellular lipid homeostasis, which most likely takes place also in the nervous system. Recently, it has been shown that WWOX directly interacts with proteins involved in protein trafficking, endosome, and lysosome networks, like SIMPLE, which were shown to cause the dominant demyelinating Charcot-Marie-Tooth neuropathy type 1C (CMT1C) [73].

In conclusion, *WWOX* gene alterations are associated with lipid metabolism. They are associated with low plasma HDL-C levels, aberrant triglyceride (TG) levels, and TG/HDL or HDL/LDL index impairment. Thus, the *WWOX* gene is therefore a promising target for future research in preventing and treating cardiovascular and neurological diseases.

## Participation of WWOX in glucose metabolism

Over the past decade, an increasing number of reports indicating the role of WWOX in the regulation of glucose metabolism have emerged [14, 15, 26, 74]. In light of the increasing incidence of sugar metabolism disorders among different human populations, gaining more knowledge about the genes and mechanisms contributing to its pathophysiology is necessary. The participation of WWOX in the control of glucose metabolism is also important in the context of its role as a tumor suppressor.

The genomic region containing the *WWOX* gene has been identified as a genetic risk factor for glucose metabolic diseases. A GWAS (genome-wide association study) of type 2 diabetes (T2DM) demonstrated that, among others, the rs7192960 genetic variant near the *WWOX* gene is associated with reduced insulin secretion [75] and that *WWOX* rs17797882 was associated with decreased HOMA-β (β-cell function indicator) in the Han Chinese population [76]. However, this was not confirmed in the Japanese population [77]. Other authors analyzed GWAS data from the Type 2 Diabetes Knowledge Portal and reported that several variants within *WWOX* are related to metabolic syndrome disorders, including T2DM [14]. Other studies have shown that the *WWOX* locus is also associated with other disorders linked to metabolic syndrome, such as obesity susceptibility [78], hypertension [79], and coronary artery calcification [80].

As a result of our observation in pregnancy-associated diabetes, we proposed that the *WWOX* gene can be an essential contributor to the pathogenesis of gestational diabetes mellitus (GDM) [15]. Decreased *WWOX* expression and, in particular, a reduction in the *WWOX/HIF1A* ratio were observed in GDM patients compared to those without GDM [15]. It is also worth mentioning that gestational diabetes patients were characterized by high *HIF1A* expression and its target genes. Hypoxia-inducible factors (HIFs) are transcription factors that play an essential role in controlling the cell response to hypoxia and take part in “the Warburg effect” [81, 82]. It was stated that WWOX, via its first WW domain, interacts with HIF1α and modulates its transactivation function [26]. The consequence of *WWOX* downregulation was the transcriptional upregulation of the glycolytic phenotype in leukocytes of patients with GDM [15]. The expression of the glucose transporter *SLC2A1* and the glycolytic gene *PFK*, *PKM2*, and *LDHA* mRNA was significantly increased in patients with GDM compared with the control subjects. Moreover, the expression of *SLC2A4*, which is an insulin-dependent glucose transporter, was significantly reduced in leukocytes [15]. We did not observe differences in *HK2* expression between the tested groups. However, the expression of *HK*2, *PFK*, *PKM2*, and *SLC2A1* correlated with *HIF1A*, but only in the GDM patient group. More importantly, the aforementioned correlations were stronger with the *WWOX/HIF1A* ratio than with the expression of *WWOX* alone. These results indicate that WWOX can modulate HIF1α activity in normal tissues in gestational diabetes patients, regulate glucose metabolism, and participate in GDM pathogenesis. Moreover, in GDM patients, a positive correlation between *HIF1A* expression and glycated hemoglobin (HbA1c), an overall glycemic marker, was also observed [15].

Our further analysis of the importance of WWOX in glucose metabolism in a human fibroblast cell line revealed that in normoxic and normoglycemic conditions, *WWOX* deficiency leads to increased *HIF1A* mRNA and protein expression. Additionally, it increases the translocation of HIF1A to the nucleus and amplifies its transactivation function [74]. The expression levels of *SLC2A1*, *ENO1*, *PKM2*, *PDK*, and *SLC2A4* were increased in *WWOX* KO cells, and insulin-dependent and insulin-independent glucose uptake, hexokinase, and lactate dehydrogenase enzymatic activities were upregulated. This clearly indicates that *WWOX* silencing leads to a shift toward anaerobic glycolysis under normoxic conditions. In terms of its importance in diabetes, studies in hyperglycemic conditions also found that, although fibroblasts are not typical insulin target cells and show little metabolic response to insulin, the level of insulin-dependent glucose uptake under hyperglycemic culture conditions was several times lower in *WWOX* KO cells than in control cells [74]. In addition, under hypoxic hyperglycemic conditions, HIF1α transactivation function was increased in *WWOX* KO cells, and increases in the expression of its target genes *PFK*, *PKM2*, and *PDK* were observed. Simultaneously, an increase in lactate concentration has been noted [74].

Initially, *WWOX* was indicated to play a role in glucose metabolism in mouse models [14, 24, 26, 28]. As mentioned above, WWOX physically and functionally interacts with HIF1α and coordinates its transactivation function in vitro and in vivo [26]. *Wwox* KO mice demonstrated higher levels of serum lactate [26]. Additionally, mouse embryonic fibroblasts (MEFs) from KO embryos [26] and muscles of mice with muscle-specific ablation of *Wwox* [14] exhibited increases in HIF1α levels and its activity, and the expression of its target genes, which encode key glycolytic enzymes, was increased [14, 26]. This clearly indicates that the downregulation of *WWOX* expression and especially a low *WWOX/HIF1A* ratio lead to a shift toward anaerobic glycolysis. Additionally, glucose uptake is increased, which is probably due to increased GLUT1 expression [26]. Moreover, *WWOX* deficiency seems to be related to increases in HIF1α expression levels and transcription factor activity, resulting in cell metabolism modifications.

Simultaneously, pyruvate dehydrogenase kinase (*PDK1*) upregulation was also observed in *WWOX*-knockout cell lines and mouse tissues [26, 74]. PDK1 is an enzyme responsible for the inactivation of pyruvate dehydrogenase, which catalyzes the conversion of pyruvate to acetyl-CoA [83]. Acetyl-CoA is a major substrate in the citric acid cycle that carries out cellular respiration. The upregulation of PDK1 levels blocks glucose influx into the tricarboxylic acid (TCA) cycle. It was also shown that *WWOX* is connected with TCA cycle inhibition. It has been shown in the *Drosophila melanogaster* model that the *WWOX* ortholog plays an important role in controlling aerobic metabolism and ROS formation [84, 85]. In this model, functional WWOX interactions with isocitrate dehydrogenase (IDH) and superoxide dismutase (SOD) were identified [84]. A significant positive correlation between WWOX and the mRNA levels of the isocitrate dehydrogenase family member *IDH1* was also observed in many human cancer cell lines [84]. Isocitrate dehydrogenase is an enzyme that catalyzes the oxidative decarboxylation of isocitrate in the TCA cycle [86], so the connection between *WWOX* and *IDH1* levels confirmed the contribution of WWOX to maintaining metabolic homeostasis. Superoxide dismutase is an enzyme that catalyzes the dismutation of superoxide radicals (O_2_^−^) to molecular oxygen (O_2_) and hydrogen peroxide (H_2_O_2_), assuring cellular protection against reactive oxygen species [87]. *SOD1* overexpression correlates with increases in *Wwox* transcripts [84]. Additionally, *Sod* mutations in flies led to changes in endogenous *Wwox* transcript levels compared with flies ectopically overexpressing human SOD1. The ectopic expression of human *SOD1* in the human HEK294 cell line also resulted in an increase in endogenous *WWOX* mRNA expression levels [84]. Ectopic *WWOX* expression in the *D. melanogaster* model led to higher ROS levels, while a decrease in *WWOX* led to a reduction in ROS levels [84]. These findings indicate that Wwox can play a protective role under conditions of oxidative stress. A *Drosophila melanogaster* model with a reduction in *Wwox* expression showed reduced mitochondrial respiration by decreasing the expression of all six mitochondrial respiratory chain genes (*ND23*, *ND42*, *ND75*, *CG7580*, *CoVa*, and *CoVb*) [88]. Then, a significant percentage of modified flies presented a phenotype with abnormal eye development [88]. It was previously reported that a reduction in the expression of these genes resulted in a visible disruption to eye morphology, which is indicative of cellular dysfunction. These phenotypes indicate that the decreased expression of mitochondrial respiratory complex genes leads to significant cellular dysfunction, so affecting mitochondrial function by WWOX deficiency is relevant [88]. However, the modification of other pathways underlying these developmental defects by WWOX cannot be ruled out. Mice with muscle-specific ablation of *Wwox* are also characterized by decreases in mitochondrial mass and TCA cycle gene expression (*Sdha*, *Ogdh*, *Mdh2*, *Idh2*, *Fh1*, *Ddlst*, and *Dlat*) [14]. Since active HIF1α inhibits the Krebs cycle [89], disruptions of the WWOX–HIF1α interaction are probably responsible for the occurrence of this phenotype.

The involvement of WWOX in metabolic syndrome was also proposed. Mice with a tissue-specific ablation of *Wwox* in skeletal muscles develop a phenotype similar to metabolic syndrome [14]. These mice exhibited hyperglycemia, obesity, and dyslipidemia. These mice also have high fasting plasma glucose and basal insulin levels, so they are deficient in their ability to take up glucose and suffer from insulin resistance [14]. Consequently, *Wwox* expression in skeletal muscles can be fundamental for maintaining glucose homeostasis. Our results suggest an even more extended, systemic effect of WWOX on insulin sensitivity. It is also interesting that an analysis of the body composition of mice with muscle-specific ablation of *Wwox* showed a significant increase in fat mass with an accompanying decrease in the lean body mass, even though food intake was decreased, compared with control mice [14]. At the molecular level, AMP-activated protein kinase (AMPK) activity was decreased in mice with *Wwox* ablation in skeletal muscles [14]. The relationship between Wwox and AMPK expression was also confirmed in a mouse myoblast cell line, so Wwox is probably a direct AMPK activator. AMPK is a key metabolic regulator known for glucose uptake stimulation and is proposed as a promising target in relation to metabolic disorders [90–92].

Moreover, type II diabetes is also a major risk factor for cognitive decline and dementia in the elderly population [93]. In Goto Kakizaki rats, which are a spontaneous model of noninsulin-dependent diabetes mellitus, an increase in activated WWOX levels was detected in the brain cortex of younger rats, while a decrease was observed in older rats [94]. In the differentiated human neuroblastoma SH-SY5Y cell line, activated WWOX was observed after 24 h of culture in high glucose [94]. Generally, glucose is the main energy substrate for the mammalian brain, and because of the high energy demand of nerve cells, neurons require continuous delivery of glucose. Neurons use glucose as a supply of the precursors for neurotransmitter synthesis and ATP for fueling their activity [95]. The principal component analysis (PCA) of global transcriptome data suggested that WWOX is involved in maintaining basic glucose metabolism in undifferentiated human neural progenitor cells (hNPC) as well as in differentiated neurons [96]. Among the genes which differentiate variants with low/high *WWOX* expression were several important genes participating in basic metabolism, such as *PDK1*, *LDHA*, *PFKP*, *HK2*, and *HIF1A* [96]. Thus, disturbed expression of *WWOX* in nerve cells most likely leads to disordered metabolic processes. Disruptions of glucose transport and metabolism impair neurons’ functionality, which is the case with neurodegenerative diseases, such as Alzheimer’s or Parkinson’s disease [97], with which WWOX has been associated. The changes of the glycolysis are seen early in neurodegenerative diseases [98], and some authors suggest that metabolic disturbances may be a root cause of neuronal loss [99]. The limitation of aerobic glycolysis in brain areas of amyloid deposition and higher accumulation of tau protein was observed in the case of Alzheimer’s disease patients [98]. Glycolysis and mitochondrial function are also decreased in Parkinson’s disease patients [100]. It has been shown that increasing glycolysis may slow neurodegeneration [100]. WWOX has been also implicated in epilepsy [61, 68]. It was observed that the anaerobic glycolysis and concentration of lactate in the epileptogenic brain increased in comparison to the control [101]. What is important, lactate can act as a signal molecule in brain cells and affect neuronal excitation [101]. The role of WWOX in the control of glucose metabolism in the context of neurological diseases requires further research.

In cancer cells, glucose metabolism is converted to fuel cell growth and division in adaptation to excessive and uncontrolled cancer cell proliferation [102]. Alterations cause changes within glucose transporters (GLUTs), enzymes of the glycolytic pathway, hypoxia-inducible factor (HIF), monocarboxylate transporters (MCTs), and lactate dehydrogenase [102]. Metabolic rearrangements lead to a preference for aerobic glycolysis by most cancer cells [102]. Aberrant glycolysis, instead of oxidative phosphorylation, in the presence of oxygen is a phenomenon called the Warburg effect [103]. *WWOX* knockdown in the MCF7 breast cancer cell line led to the upregulation of HIF1α glycolytic genes. Additionally, in breast cancer samples, WWOX expression negatively correlated with GLUT1 levels, which supports the hypothesis that *WWOX* modulates cancer glucose metabolism [26]. Furthermore, in a rescue experiment in MCF7 breast cancer cells, the upregulation of *WWOX* suppressed HIF1α target gene expression [26].

## Influence of WWOX on bone metabolism

Apart from the aforementioned defects, the homozygous deletion of *Wwox* in mice caused disturbances in bone metabolism, such as osteopenia [24]. The decrease in trabeculae bone density along with the thinning of the inner cortex led to the development of disproportionately smaller limbs in *Wwox*-deficient mice [24]. Osteoblasts isolated from *Wwox*-deficient mice exhibited disturbances in differentiation, beginning at the mineralization stage [24]. Additionally, an increase in osteoclast activity was observed [24]. In *Wwox* KO mouse osteoblasts, it was shown that the expression of markers of the early stage (Runx2, Alp), matrix production (Bsp, ColI), and mineralization stage (Oc) significantly declined, so they could not produce sufficient bone matrix to compensate for bone loss [24]. Microcomputed tomography analyses showed significantly decreased parameters of bone formation, such as changes in bone growth, density, formation, and resorption characteristics in *Wwox*^−/−^ mice [24]. Moreover, measurements showed a 50% reduction in serum calcium and a 20% increase in serum phosphate concentration in *Wwox*-deficient mice [24]. Calcium and phosphate metabolism and the maintenance of their homeostasis are key for physiology and skeletal mineralization [104]. Interestingly, in *Wwox* KO mice, the upregulation of thyroid hormone was identified, which is known to contribute to metabolic bone disease [24].

The principal transcriptional regulator involved in osteoblast differentiation is RUNX2 [105–107]. It was shown that WWOX physically interacts with RUNX2 [24]. This inhibits the transcription factor function of RUNX2, leading to the repression of many RUNX2 targets involved in bone matrix formation [24]. In *Wwox* KO mice, *Runx2* expression was increased in calvarial and femoral bones [24]. Therefore, WWOX is able to regulate RUNX2 at two levels: as an indirect inhibitor of *RUNX2* expression and as a suppressor of RUNX2 transcriptional activity.

Nearly one-third of *Wwox* KO mice develop osteosarcomas [28]. In human osteosarcomas, WWOX expression is reduced, and a positive correlation between *WWOX* expression and patient response to chemotherapy was found [108]. In a human osteosarcoma cell line model, the ectopic expression of *WWOX* inhibited proliferation and attenuated invasion in vitro and suppressed tumorigenicity in mice [109]. Most likely, the disruption of the WWOX–RUNX2 interaction is crucial for osteosarcoma development. *RUNX2* mRNA and protein levels increase in bony tissues of *Wwox*-deficient mice [109]. It was also found that high *WWOX* expression was associated with reduced *RUNX2* expression in osteosarcoma cell lines [109]. An inverse WWOX and RUNX2 interaction was not as clear in human osteosarcomas as in cell lines. Thus, this relationship may be more complicated in in vivo tumors due to the complexity of various further aberrations [110]. The suppression of the transactivation function of RUNX2 by WWOX probably contributes to the tumor suppressor role of WWOX. It is also meaningful that *RUNX2* is overexpressed in breast and prostate cancer metastases to bone [111, 112].

## Conclusions

**Fig. 1:**
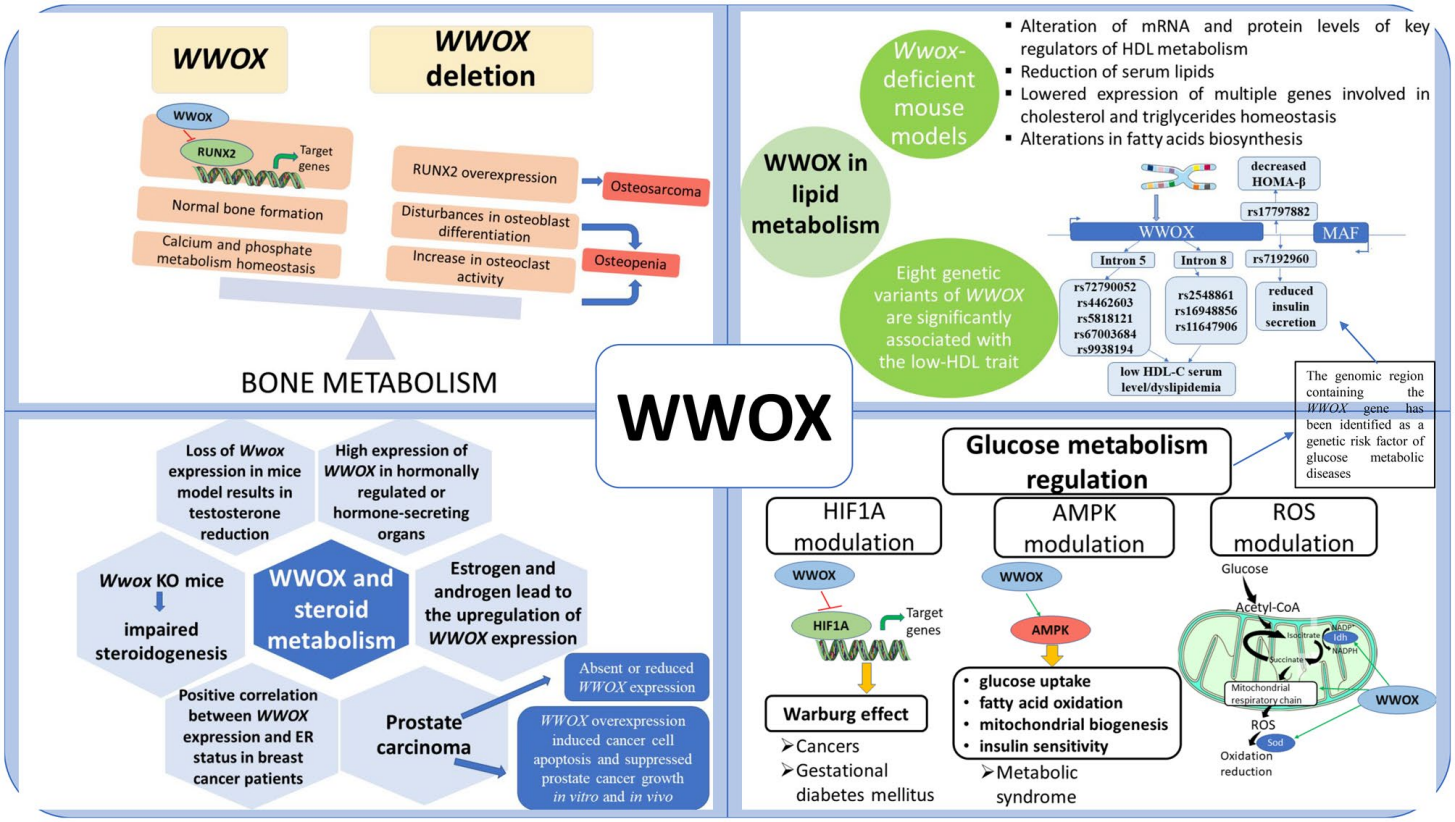
Schematic model of information about the WWOX role in metabolism

In this review, we have attempted to emphasize what we consider to be important knowledge about the role of the *WWOX* gene in metabolism. There is growing evidence of the tumor suppressor activity of WWOX in a number of different cancers. Moreover, *WWOX* germline mutations were recognized as the cause of severe developmental pathologies of the brain, such as autosomal recessive spinocerebellar ataxia 12 (SCAR12) and WWOX-related epileptic encephalopathy (WOREE syndrome). In addition to typical tumor suppressor functionalities related to proliferation or invasion, WWOX-related metabolic changes have been observed in many different cancers. Several reports have indicated the role of WWOX in the regulation of steroid, cholesterol, glucose, and bone metabolism, which is summarized in Fig. 1.

WWOX binding is capable of binding to hundreds of different proteins, including many transcription factors (TF), thereby modulating the transcription of a wide variety of genes. One of the most important known partners of WWOX is TFs, such as HIF1α and RUNX2. WWOX is involved in the control of the transcriptomic activity of these factors; therefore, *WWOX* gene alterations are associated with multiple metabolic abnormalities. One of particular interest is the involvement of WWOX in the pathology of glucose and basic energy metabolism. WWOX participates in the induction of the Warburg effect in cancer or the pathology of gestational diabetes and diabetes type II. Conceivably, the partnership between WWOX and HIF1α is crucial in the regulation of tumor metabolism and diabetes pathology. WWOX can play a protective role by controlling HIF1α activity, and it makes the influx of glucose into mitochondria possible. Therefore, it is able to correct the operation of the Krebs cycle and thus prevents the Warburg effect. Moreover, WWOX is associated with pathologies related to mineral metabolism, including bone formation and calcification. Proper WWOX protein structure and concentration are crucial requirements in postnatal survival, growth, and metabolism and probably play an essential role in the regulation of bone tissue formation. According to the current knowledge, all these functions and disorders are realized through the WW domain through which WWOX binds to a number of proteins to modify their action. Furthermore, mouse models and human genetic studies have established a significant physiological role for WWOX in lipid and lipoprotein metabolism. WWOX is probably involved in the complicated interactions that maintain cholesterol homeostasis and is an important regulator of HDL and TG levels, in both humans and mice.

WWOX is a hub protein that globally regulates gene expression by modifying TF activity in particular, and it is one of the crucial molecular elements of cell functioning. Specific pathologies associated with mutations, genetic variants, and differential WWOX transcription are dependent on the type of cell and tissue, the stage of differentiation, and the development or physiological state. Thorough understanding of the complex mechanisms of action of the WWOX protein will surely provide an understanding of many pathologies and hopefully pave the way for new therapies. Thus, accumulating reports demonstrating the potential role of WWOX in many types of metabolic disorders and metabolic rearrangements in cancer opens the possibility for its therapeutic implementation while also contributing to our understanding on a basic science level.

## Data Availability

Not applicable.
